# Facile synthesis and characterization of ZnO nanoparticles grown on halloysite nanotubes for enhanced photocatalytic properties

**DOI:** 10.1038/s41598-017-02501-w

**Published:** 2017-05-22

**Authors:** Hongxia Peng, Xiaohe Liu, Wei Tang, Renzhi Ma

**Affiliations:** 1 State Key Laboratory of Powder Metallurgy and School of Materials Science and Engineering, Central South University, Changsha, Hunan P.R. China; 2 Hunan Provincial Key Laboratory of Fine Ceramics and Powder Materials, Hunan University of Humanities, Science and Technology, Lou’di, Hunan P.R. China; 3 International Center for Materials Nanoarchitectonics (MANA), National Institute for Materials Science (NIMS), Namiki 1-1, Tsukuba, Ibaraki 305-0044 Japan

## Abstract

We demonstrated herein that ZnO nanoparticle with sizes in the range of 3–5 nm grown on the surface of halloysite nanotubes (HNTs) could be facile prepared in large quantities through the seed-mediated growth process using ZnAc_2_·2H_2_O as the zinc source. Compared with the individually dispersed ZnO nanoparticles, the as-prepared HNTs@ZnO nanocomposites showed a smaller band gap energy and relatively strong light absorption. Therefore, HNTs@ZnO nanocomposites possessed higher photocatalytic activity than individually dispersed ZnO nanoparticles, exhibiting the HNTs@ZnO nanocomposites could be used as highly efficient photocatalysts. The HNTs@ZnO nanocomposites endowed HNTs special performance and improve the catalytic activity of ZnO, which originated from narrow band gap and chemical passivation induced by a negative fixed charge in the HNTs support.

## Introduction

During earth’s evolution, the changing environment provided suitable conditions for the formation of natural nanomaterials. Silicate minerals, such as montmorillonite, palygorskite and halloysite are pervasive compounds in the earth’s crust and often exist in nano-size or in various nano-morphologies^1–4^. With the development and progress of society, the importance and value of natural nanomaterials in science has been acknowledged. These nanomaterials have the potential to be used as inexpensive alternatives to expensive carbon nanotubes or other nanomaterials for various applications^5, 6^. Among these natural nanomaterials, halloysite (Al_2_Si_2_O_5_(OH)_4_·2H_2_O), with excellent physical and chemical properties, environmental friendliness and high chemical stability has been extensively investigated in different domains^7–9^. Halloysite nanotubes (HNTs) are naturally occurring alumino-silicate minerals with a well-defined tubular nanostructure, which consists of outside-in alternate silica tetrahedron sheets and alumina octahedron sheets in a 1:1 stoichiometric ratio^10–12^. However, HNTs with high chemical stability results in a difficulty in wide application. Some studies have shown that coupling HNTs with semiconductors or metals to form composite photocatalysts can expand them the potential application fields by adding new functionality. Cheng *et al*. synthesized TiO_2_/HNTs composite photocatalyst by loading N-doped TiO_2_ on HNTs, in which the TiO_2_ worked as an active site to degrade pollutants^13^. Wang *et al*. synthesized Pt@RHNTs composite catalyst by loading Pt on HNTs, which shows rapid catalytic rates in hydrogenation reactions and excellent leaching resistance in cycle uses^14^. Similarly, other materials such as Fe_3_O_4_, MnO_2_, La_0.7_Ce_0.3_FeO_3_ and graphene oxide in the composite also can act as an active site to HNTs added magnetic and photocatalytic activity^15–17^.

ZnO has gained more and more attention in the field of environmental purification for its wide band gap (3.3 eV), large excitation binding energy of 60 meV, high catalytic activity, and environmental friendliness^18, 19^. The photocatalytic activity of ZnO is dramatically higher than that of TiO_2_ light photocatalysts. In particular, compared with TiO_2_, ZnO is easily obtained at a lower cost, and more advantageous to realize large-scale industrialized production^20–22^. Unfortunately, ZnO nanoparticles are prone to aggregate because large excitation binding energy of 60 meV, which results in an adverse effect on the photocatalytic activity. And the effective utilization rate and adsorption rate of organic pollutants of ZnO is lower. Therefore, ZnO nanoparticle assembled on HNTs is promising to simultaneously possess excellent photocatalytic activity and absorptivity, meanwhile to improve effective utilization rate of ZnO and HNTs, which could deliver exceptional performances in photocatalytic degradation of pollutants. Cheng *et al*. synthesized ZnO encapsulated in HNTs catalysts by using the impregnation method, which showed highly solar-light photocatalytic activity compared to pure ZnO nanoparticles^23^. But ZnO nanoparticels was capsulated into HNTs tube, reduces the light absorption ability of ZnO.

Herein, a novel kind of HNTs@ZnO nanocomposites was fabricated via a seed-mediated growth process. The motivation for designing HNTs@ZnO nanocomposites was due to the following aspects: the first is to expand the new function of HNTs (photocatalytic activity); the second is to block the aggregation of ZnO nanoparticles, meanwhile to reduce electron-holes recombination rate of ZnO surface and band gap, thus improving the photocatalytic activity; the third is to reduce the amount of ZnO, and improve effective utilization rate of ZnO; the fourth is to achieve pre-concentration of pollutants and improve the separation of nanosized active particles by using HNTs as a support. Furthermore, the photocatalytic activity and stability, as well as the photocatalytic mechanism of the HNTs@ZnO nanocomposites for decomposing methylene blue (MB) were investigated. Compared with ZnO and HNTs, the HNTs@ZnO nanocomposites exhibited excellent photocatalytic activity and adsorption performance, which endowed the nanocomposites with a bright perspective in degradation of organic pollutant. In addition, current synthetic strategy could be used to synthesize other HNTs nanocomposites under appropriate condition, and it has promising prospects for future large-scale applications owing to its high yields, simple reaction apparatus, and mild conditions.

## Results

**Figure 1:**
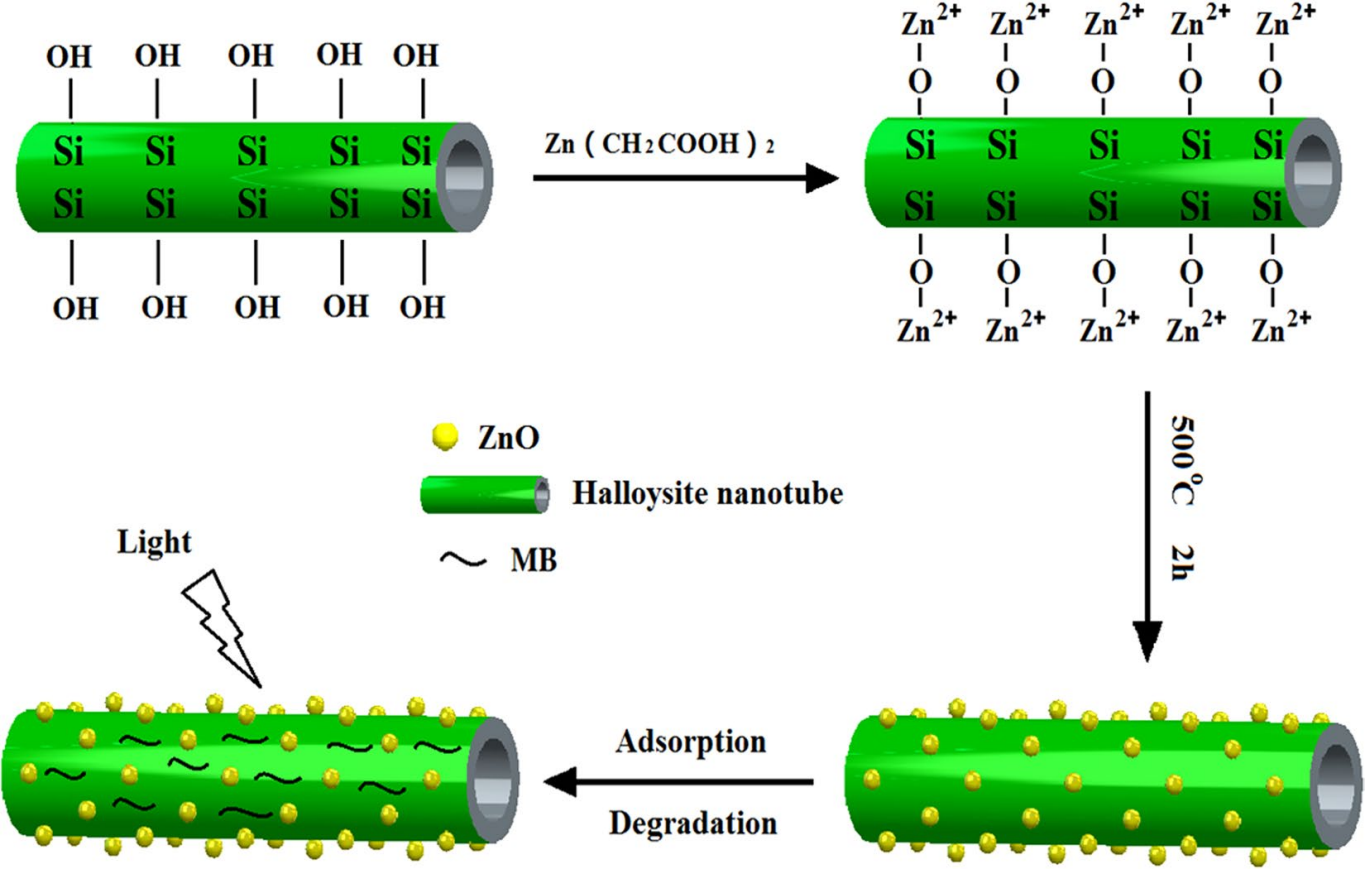
Illustration of the formation process of HNTs@ZnO nanocomposites.

The seed-mediated growth method has been considered as a simple and cost-effective preparation approach to obtain nanocomposites. In the present study, we used this technique for synthesized HNTs@ZnO nanocomposites through ZnO successfully deposited on HNTs surface, inspiring by the surface hydroxyl group of the HNTs, which is connect with Zn^2+^ ions via an O-Zn^2+^ coordination bond at room temperature^24^. The product was calcinated for 2 h at 500 °C to prepare HNTs@ZnO nanocomposites, as shown in Fig. 1.

**Figure 2:**
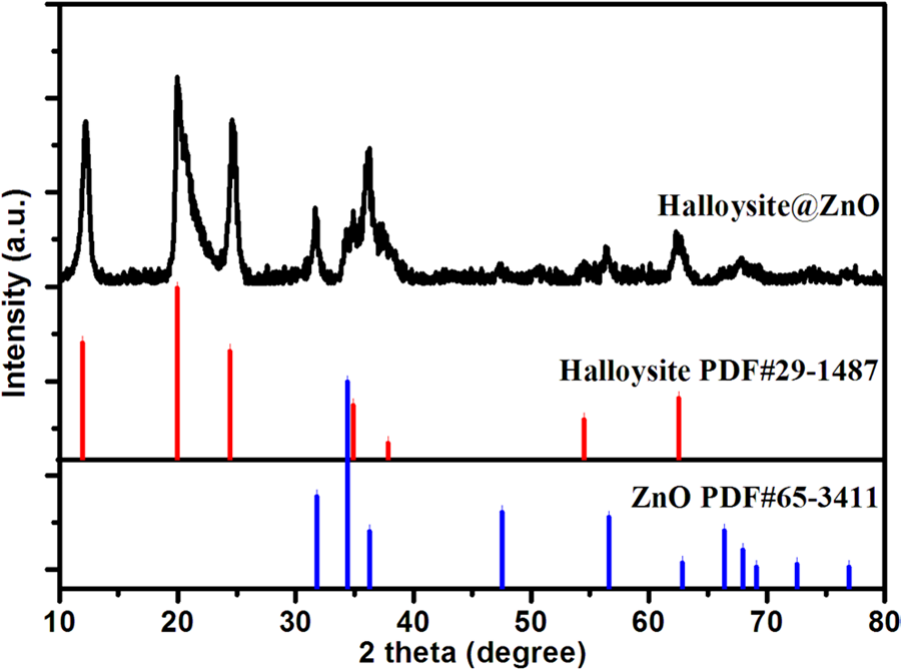
XRD patterns of as-prepared HNTs@ZnO nanocomposites.

X-ray diffraction (XRD) was carried out to investigate the structure and composition of the HNTs@ZnO nanocomposites as shown in Fig. 2. In the case of HNTs@ZnO, besides the characteristic diffractions of monoclinic system HNTs (PDF 29-1487), the obvious diffraction peaks at 2*θ* = 31.68°, 34.38°, 36.25°, 47.53° and 68.03° can be indexed to the tetragonal phase of ZnO (PDF 65-3411), which suggested the successful crystallization of ZnO on the surface of HNTs. Additionally, no additional peaks for other phases can be detected, which indicated that no reaction occurred between core and shell during the synthesis process.

**Figure 3:**
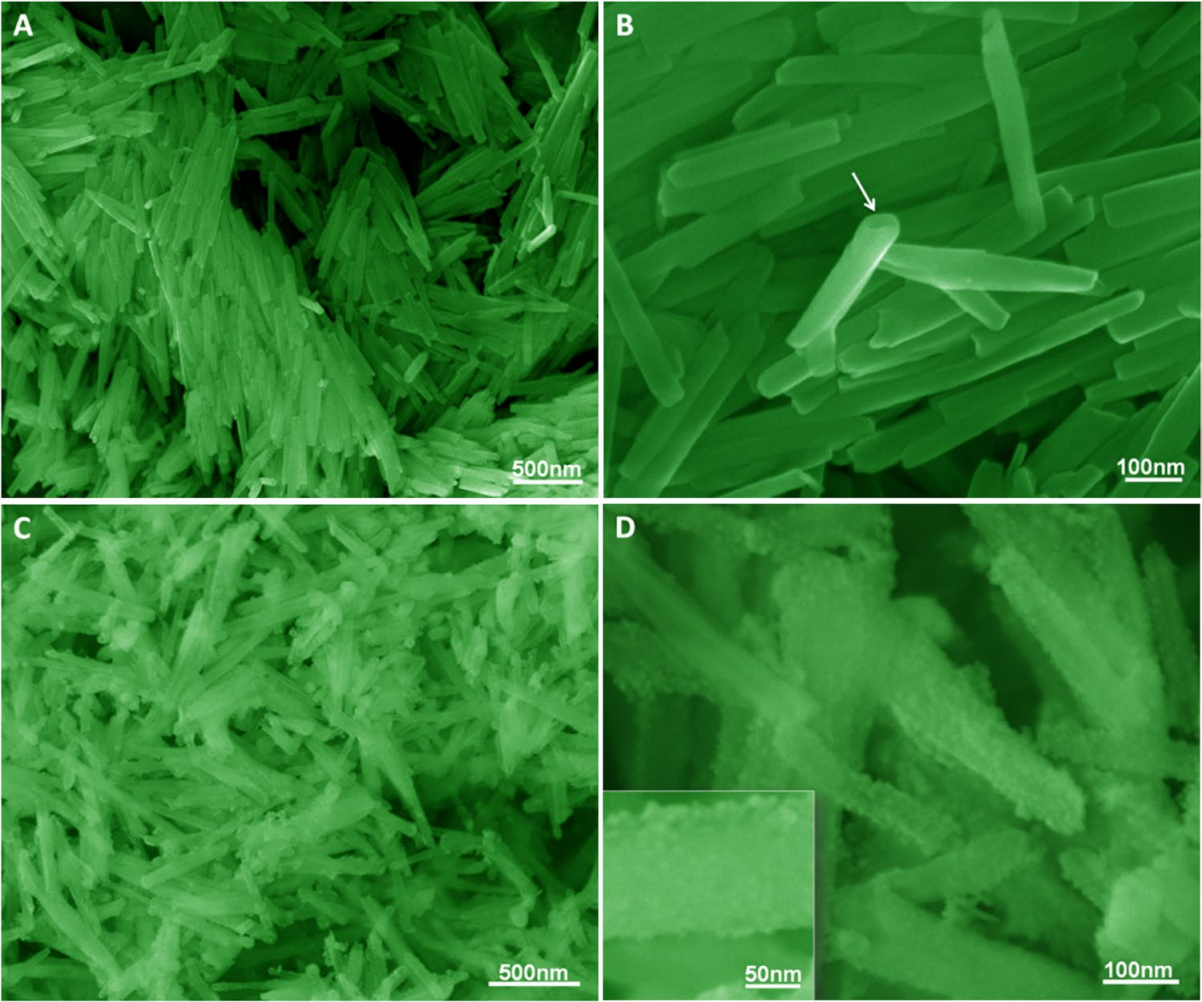
SEM images of (**A**,**B**) HNTs and (**C**,**D**) HNTs@ZnO nanocomposites. The inset in (**D**) shows a high-magnification SEM image.

The structure and morphology of HNTs and HNTs@ZnO nanocomposites were characterized by scanning electron microscopy (SEM). As shown in Fig. 3A and B, the HNTs consist of cylindrical tubes with diameter in the range of 30–50 nm and length of up to 1 μm. SEM image reveals the typical tubular structure of HNTs (see arrow in Fig. 3B) with smooth surface. After surface modification by coating the ZnO shell, the HNTs@ZnO nanocomposites retained their cylindrical tubular morphology with a lack of aggregation (Fig. 3C,D). The color of the HNTs@ZnO nanocomposites became lighter. The ZnO nanoparticles are coated on the surface of HNTs nanotubes, which makes the surface of HNTs come more rough and loosen (Fig. 3D). These results suggest that the ZnO nanoparticles were coated on the surface of HNTs nanotubes.

**Figure 4:**
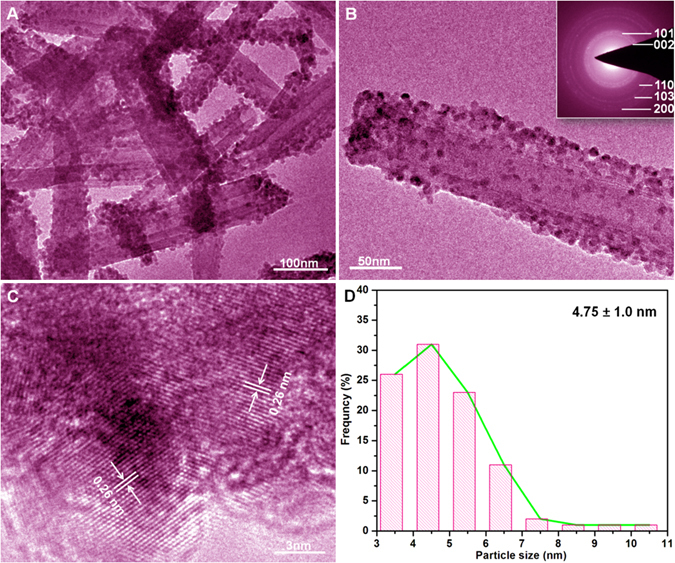
(**A**,**B**) TEM and (**C**) HRTEM images of HNTs@ZnO, (**D**) ZnO particle-size histogram.

The morphologies of as-prepared HNTs@ZnO nanocomposites were characterized using transmission electron microscopy (TEM) and high-resolution TEM (HRTEM), as shown in Fig. 4. The HNTs@ZnO nanocomposites exhibit a typical tubular nanostructure with an outer diameter and inner diameter of approximately 10 nm and 5 nm, respectively (Fig. 4A,B). The inset shown in Fig. 4B is the SAED pattern taken from the HNTs@ZnO samples, which shows polycrystalline diffraction rings consisting of discrete diffraction spots. The diameters of the diffraction rings were measured and calculated. It was found that the diffraction rings correspond well to the hexagonal ZnO, this result is in well agreement with the above XRD. The above observations demonstrate that HNTs@ZnO core-shell nanoparticles are successfully fabricated by the method used in this work.

The HRTEM image of HNTs@ZnO nanocomposites (Fig. 4C) clearly demonstrates that these nanoparticles are hemispherical, and bind tightly on the surface of HNTs. Their lattice spacing was observed to be about 0.26 nm, which corresponds to the (002) plane of ZnO, confirming that these nanoparticles are ZnO crystals^24^. It is inferred that HNTs plays an important role during the *in situ* growth of ZnO particles on the HNTs surface. In the HNTs@ZnO nanocomposites, many nanoparticles with sizes in the range of 3–5 nm (Fig. 4D) are uniformly coated on the HNTs surface. The Zn^2+^ ions are firstly adsorbed on the negatively-charged HNTs surface, and then nucleation and growth of ZnO occur on the HNTs surface after being calcinated at 500 °C for 2 h (Fig. 1). Due to the surface confinement of HNTs, the growth and aggregation of ZnO are inhibited, thus leading to the formation of uniform ZnO nanoparticles^25^.

In addition, HNTs exhibits distinct lattices, indicating that it is well-crystallized after calcination at 500 °C for 2 h. It is noteworthy that no bare HNTs or peeled-off ZnO nanoparticles were observed, even though the HNTs@ZnO nanocomposites were treated ultrasonically for 1 h prior to the TEM characterization. Herein, it is speculated that the ZnO nanoparticles form covalent bonds with HNTs after the *in situ* growth process, rather than weak physical interactions.

**Figure 5:**
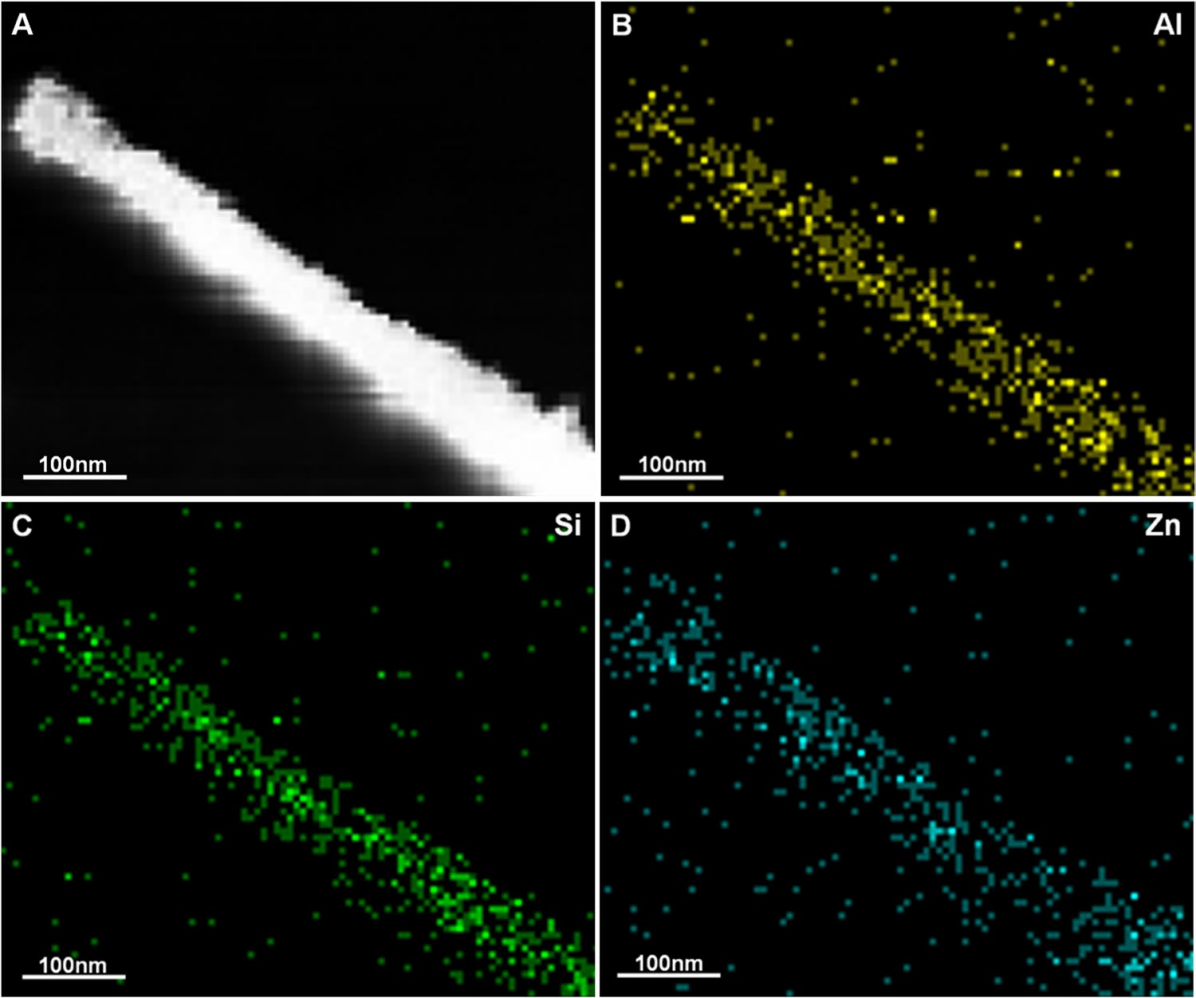
(**A**) TEM images of HNTs@ZnO nanocomposites; (**B**–**D**) energy dispersive X-ray (EDX) mapping of HNTs@ZnO nanocomposites.

To further identify the structure of the HNTs@ZnO nanocomposites, the corresponding EDX mapping of HNTs@ZnO sample was detected to analyze the elements, as shown in Fig. 5(A–D). Figure 5(B–D) represent the mapping of Al, Si, and Zn elements, respectively. It can be seen that all the elements were distributed homogeneously in the sample. This indicates that the ZnO nanoparticles were diffused after annealing at 500 °C. From EDX mapping and TEM, we can further confirm the formation of HNTs@ZnO nanocomposites.

**Figure 6:**
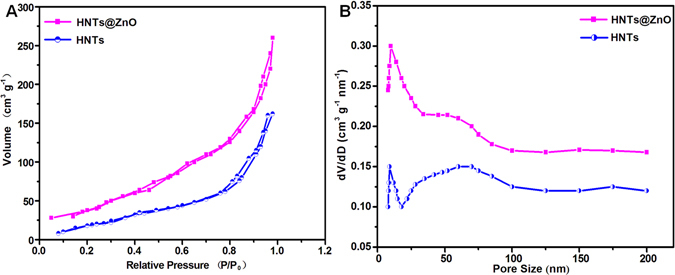
N_2_ adsorption–desorptionisotherms and pore size distributions of ZnO@HNTs and HNTs.

To further determine the surface structure of ZnO@HNTs and HNTs, N_2_ adsorption–desorption measurements were employed. Figure 6 illustrated N_2_ adsorption/desorption isotherms and pore size of the ZnO@HNTs and HNTs. As shown in Fig. 6A, all of the isotherms showed a typical type IV hysteresis loop as defined by IUPAC. The temporary locking of liquid N_2_ and the delayed evaporation in the desorption isotherm indicated that the product behaves similarly to the type H3 hysteresis loops, implying the abundant presence of hierarchical pores. It can be seen that ZnO@HNTs exhibited the largest surface area (55.3 m^2^ g^−1^) much higher than that of HNTs (44.9 m^2^ g^−1^) due to the surface of HNTs is loaded on ZnO nanoparticles with small size, which makes its the specific surface increase. The pore distribution obtained using the Barrett–Joyner–Halenda (BJH) theory indicated the porosity characteristics of ZnO@HNTs and HNTs (Fig. 6B). From the Fig. 6B, the pore size of HNTs is mainly distributed in about 12 nm and 60 nm, corresponding to the cylindrical hole of the nanotube and the accumulation hole. In the pore size distribution of ZnO@HNTs, the strong peaks appeared at 17 nm, and the peak of accumulation hole decreased, indicating that the ZnO@HNTs formed a more concentrated and uniform pore size distribution. The pore volume of HNTs and ZnO@HNTs was 0.20 cm^3^/g and 0.37 cm^3^/g, respectively.

**Figure 7:**
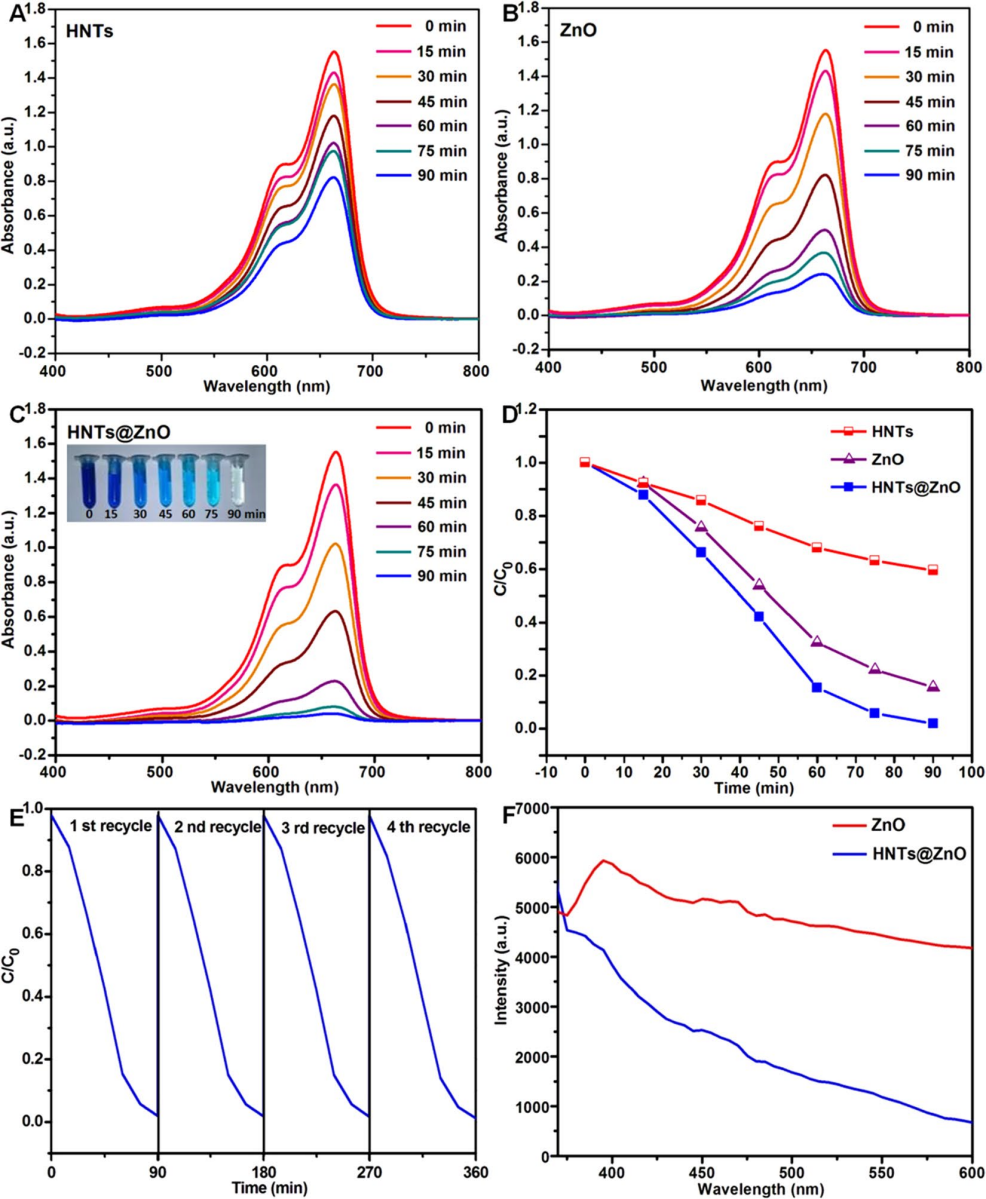
(**A**) Photocatalytic degradation curves of MB over HNTs, ZnO and HNTs@ZnO under ultraviolet light, and (**B**–**D**) the corresponding kinetic simulation curves. The Inset in (**C**) shows the corresponding solution color. (**E**) Effect of cycles of HNTs@ZnO nanocomposites on degradation of MB. (**F**) Photoluminescence (PL) spectra of ZnO and HNTs@ZnO.

The photocatalytic activities of HNTs, ZnO and HNTs@ZnO nanocomposites were carried out for decomposing MB under ultraviolet-light irradiation, and the results are shown in Fig. 7. Figure 7 shows the degradation of 20 ml MB solution on HNTs, ZnO and HNTs@ZnO nanocomposites under simulated light irradiation. After keeping HNTs dispersed in MB solution in the light irradiation for 90 min, the concentration of MB solution decreases about 40%, which should be ascribed to the absorption of MB on the HNTs. Compared to HNTs, ZnO exhibits a certain photocatalytic activity for MB. After 90 min of irradiation time, the maximum degradation rate reaches 85%, which is close to nine times higher than that of HNTs. Surprisingly, the HNTs@ZnO nanocomposites put up a remarkably higher photocatalytic activity for degradation of MB after starting light source for 60 min, reaching 78%.

As shown in Fig. 7, the degradation rate of MB over the HNTs@ZnO nanocomposites reaches 99% after 90 min of irradiation time, which is ten times and 1.2 times higher than those of HNTs and ZnO, respectively. The maximum degradation rate of MB over the HNTs@ZnO nanocomposites is up to 99%, exhibiting an excellent light photocatalytic activity. Figure 7(B,C,D) shows the UV-vis absorption spectra of MB over HNTs, ZnO and the HNTs@ZnO nanocomposites after different irradiation times. As easily observed, the typical UV-vis absorption peak of MB is 665 nm in UV-vis spectra. In contrast to MB, the height of the UV-vis characteristic absorption peak of MB solution treated by HNTs with 90 min of ultraviolet-light irradiation times lightly decreases about 40%, which is close to the absorption value above. MB solution under UV-vis irradiation about 90 min in the presence of ZnO catalysts, the height of the typical UV-vis absorption peak of MB almost decreases about 85%. However, after UV-vis irradiation about 90 min, the absorption bands of MB solution with HNTs@ZnO nanocomposites have nearly disappeared. These results are consistent with the degradation rate above. These results illustrate that most of MB over HNTs@ZnO nanocomposites was decolorizated and degradated relying on active hydroxyl free radical oxidation (**·**OH), it will chromophoric group hydrophobic (-s-) oxidation to sulfone group (absorption wavelength <180 nm). The excellent photocatalytic activity of the HNTs@ZnO nanocomposites may be ascribed to the combination effect of the ZnO nanoparticles and HNTs support as adsorbent, i.e. integrated photo-catalytic adsorbents. Most importantly, HNTs reduced the recombination rate of ZnO surface electron–holes and band gap, the immobilization of ZnO active nanoparticles on HNTs to create novel integrated photo-catalytic adsorbents is more promising for practical application in the removal of harmful organic compounds in wastewater.

Although the as-prepared HNTs@ZnO nanocomposites exhibit higher light-driven photocatalytic activity, the stability during photocatalytic reactions is also important in view of its applications. To evaluate the stability and efficiency of the HNTs@ZnO nanocomposites, the circulating runs in the photocatalytic degradation of MB under light irradiation were also investigated. As shown in Fig. 7E, it can be seen that the photocatalytic activity of the HNTs@ZnO nanocomposites do not show any obvious loss after four recycles for the photodegradation of MB. It suggests that the nanocomposites are stable and had not been photo-corroded during the photocatalytic degradation of the model dye molecules, which is very important for its practical applications.

To further investigate the role of HNTs support to the ZnO performance, we carried out the photoluminescence (PL) experiments with a 350 nm pulsed laser as excitation source. PL spectra are often used to study the surface processes involving electron–hole recombination of semiconductors. The broad band emission around 395 nm, as shown in Fig. 7F, can be assigned to the recombination of photo-excited holes with electrons occupying the singly ionized oxygen vacancies in ZnO^26–28^. Apparently, the PL intensity of the HNTs@ZnO decreases remarkably compared with ZnO, which can be ascribed to the reduction of the recombination process after the HNTs as support.

## Discussion

**Figure 8:**
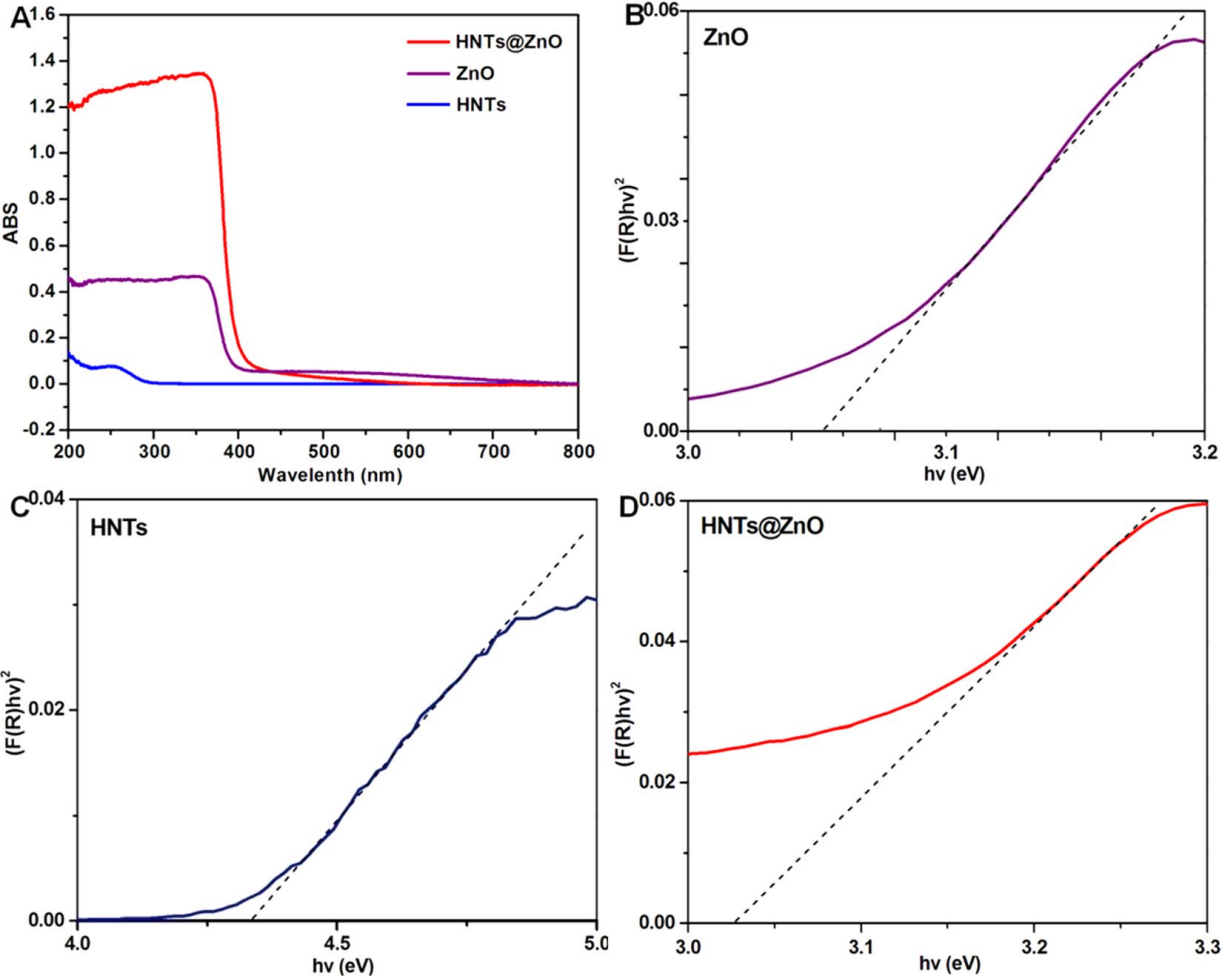
(**A**) UV-vis spectra of HNTs, ZnO and ZnO/HNTs; (**B**–**D**) DRS spectra of ZnO, HNTs and HNTs@ZnO with the corresponding plots of [F(R∞)hv]^2^ versus hv.

The UV-vis diffuse reflectance spectra (DRS) of HNTs, ZnO and HNTs@ZnO nanocomposites are displayed in Fig. 8A. It can be seen that the individually dispersed ZnO nanoparticels can absorb UV light with an absorption edge about 354 nm, corresponding to a band gap energy of 3.053 eV (Fig. 8B), which agrees with the light-absorption properties of ZnO nanoparticels reported in the literature^29^. In contrast to HNTs, the HNTs@ZnO nanocomposites appear as an obvious absorption band located at around 361 nm, which is clearly confirming that the ZnO modification can endow the optical properties to HNTs. Compared to that of individually dispersed HNTs (4.338 ev, Fig. 8C), the absorption edge of HNTs@ZnO heterojunction has an obvious red shift of about 100 nm, which can be attributed to the heteroepitaxial growth of ZnO on the HNTs surface^30^.

The band gapenergy (Eg) of the resulting HNTs@ZnO nanocomposites can be estimated from a plot of *[F(RN)hn]* ^*1/2*^ versus *hn*, where *R* is the reflectance coefficient, *h* is Planck’s constant and *n* represents the light frequency. The tangent interception with the *X* axis gives a good approximation of *Eg* for the HNTs@ZnO nanocomposites. The band gaps of the HNTs@ZnO nanocomposites are about 3.026 eV (Fig. 8D). Better than ZnO nanoparticles and HNTs, HNTs@ZnO nanocomposites with a narrow band gap should have a high photo-catalytic activity for target reactions. But band gap modification is not really significative, which explain that HNTs almost have no effect to band gap of ZnO. The difference in band gap might attribute to the content of oxygen vacancies. Wang *et al*. reported that when the concentration of oxygen vacancies is increased, the impurity states become more delocalized and overlap with the valence band edge, resulting to the band gap narrowing^31^.

**Figure 9:**
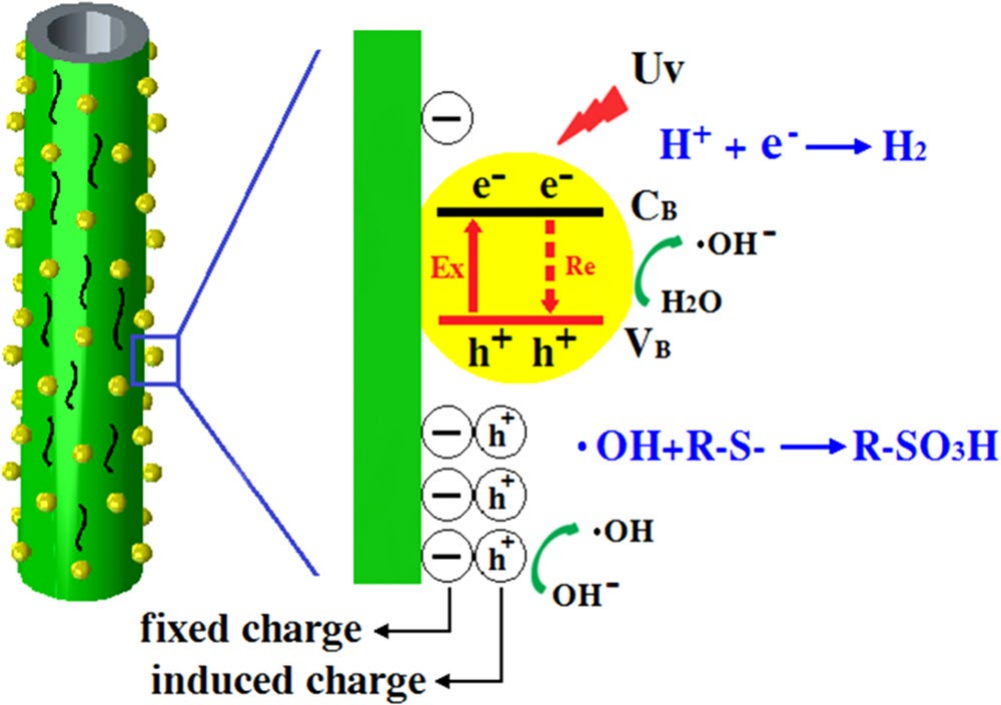
Schematic illustration of the photocatalytic degradation mechanism of MB over HNTs@ZnO nanocomposites under UV-light.

On the basis of the above experimental results and previous reports^32^, it is deduced that the unusual high catalytic efficiency of the HNTs@ZnO photocatalyst originates from the chemical passivation and absorptivity of HNTs support. The photocatalytic mechanism is proposed in Fig. 9. The left section of the structure showed that the ZnO deposited on the surface of HNTs. The right section demonstrated the Energy band diagram of ZnO coated on the surface of HNTs, where VB and CB are the valence and conduction bands, respectively. Under visible-light irradiation, the photo-generated electrons are excited from the valence band (VB) to the conduction band (CB) in ZnO, inducing the formation of holes in the VB. The photo-generated holes in the VB of ZnO can quickly transfer to the interface of ZnO and HNTs support because of the presence of the negative charges that localized in the HNTs support close to the interface with ZnO. Photogenerated holes are trapped at the surface due to the presence of the negative charges located in HNTs support, leaving behind unpaired electrons on the surface of ZnO. As a result, both the holes transferring to the surface of HNTs support and those remaining in the VB of ZnO can be employed for the subsequent oxidation reactions of MB. Such coupled mechanisms (chemical passivation) will spatially separate the photo-generated electron-holes with suppressed photo-carrier recombination and dramatically improve the photocatalytic activity of HNTs@ZnO nanocomposites.

## Conclusions

In summary, a seed-mediated growth approach was developed to prepare ZnO-decorated HNTs. The as-prepared ZnO-decorated HNTs showed a significantly red shifted when compared with the individually dispersed HNTs nanoparticles, which can be attributed to the heteroepitaxial growth of ZnO on the HNTs surface. And as compared with individually dispersed ZnO nanoparticles, the as-synthesized ZnO-decorated HNTs exhibited superior photocatalytic performance toward MB photo-degradation, attributable to the conspicuous light absorption of the decorated ZnO that can induce the generation of abundant active hydroxyl free radical oxidation (·OH) for participation in MB degradation. Furthermore, the adsorbability of HNTs surface played critical roles both in the MB degradation process. The findings from this work also sheds light on the development of composite HNTs/semiconductor photo-catalyst systems in which light mediation synergistically regulate the carrier generation and charge transfer to enhance the photoactivities.

## Methods

### Materials and Reagents

The halloysite nanotubes (HNTs) were obtained from Xin Cheng Shen Fei Aluminum Alloy Co., Ltd. (Wenzhou, China). Zinc acetate (ZnAc_2_·2H_2_O, AR) was purchased from the National Reagent Corporation (Shanghai, China). All reagents were analytical grade and used without further purification.

### Preparation of HNTs@ZnO Nanocomposites

In the typical preparation, ZnAc_2_·2H_2_O (1 g) was dissolved in 40 mL of deionized water to obtain the precursor solution. Then halloysite nanotubes (0.5 g) were added to the above solution under constant stirring for 24 h. After the reaction was completed, the precipitates were washed with deionized water and ethanol several times and dried in a vacuum oven at 60 °C for 4 h. The dried precipitates were then calcinated for 2 h at 500 °C. The resulting products were HNTs@ZnO nanocomposites.

### Photocatalytic Activity

The photocatalytic performance of HNTs@ZnO nanocomposites was evaluated by monitoring the photodegradation of methylene blue (denoted as MB) and the as-synthesized products were magnetically stirred thoroughly in the dark until reaching the adsorption equilibrium of MB on catalyst before exposure to UV irradiation providing a 250 W high-pressure Hg lamp (centered at 365 nm). A quartz tube with a capacity of 20 mL was used as the photoreactor vessel. Three kinds of materials, including ZnO nanoparticles, HNTs, and HNTs@ZnO nanocomposites were used and compared in the photodegradation of MB. A typical experiment involved adding 10 mg of photocatalyst to 20 mL of MB aqueous solution (10 mg L^−1^) in the photo-reactor vessel. Prior to irradiation, the suspension was aerated and stirred in the dark for 30 min to reach the adsorption equilibrium of MB with photocatalyst. At certain time intervals of irradiation, 2 mL aliquots of reaction solution were withdrawn and centrifuged to remove the photocatalyst particles. The UV-visible absorption spectra of the filtrates were then acquired to measure the concentration variation of MB by recording the corresponding absorbance of the characteristic peak at 665 nm. The concentration decay data were then fitted using a pseudo-first-order model to determine the apparent rate constant of MB photodegradation.

### Characterization

The morphology and microstructure of as-prepared products were analyzed with a SIEMENS Diffraktometer D5000 X-ray diffractometer. ULTRA-55 field emission scanning electron microscopy (FE-SEM) and transmission electron microscopy (TEM, JSM-2100) equipped with an energy dispersive X-ray spectrum (EDS, Inca Energy-200) at an accelerating voltage of 200 kV. The light absorption properties were measured using an UV-vis diffuse reflectance spectrophotometer (Hitachi, U-3010) with a wavelength range of 200–800 nm, and BaSO_4_ was used as a reference. UV-visible absorption spectra were collected with a Hitachi U-3100H spectrophotometer at room temperature under ambient conditions.
